# Gut microbiota profile and the influence of nutritional status on bacterial distribution in diabetic and healthy Tunisian subjects

**DOI:** 10.1042/BSR20220803

**Published:** 2023-09-12

**Authors:** Meriem Fassatoui, Azadeh Saffarian, Céline Mulet, Henda Jamoussi, Amel Gamoudi, Yosra Ben Halima, Meriem Hechmi, Sonia Abdelhak, Abdelmajid Abid, Philippe J. Sansonetti, Thierry Pedron, Rym Kefi

**Affiliations:** 1 Laboratory of Biomedical Genomics and Oncogenetics, Institut Pasteur de Tunis, Tunis, Tunisia; 2 University of Tunis El Manar, Campus Universitaire Farhat Hached, Tunis, Tunisia; 3 Unité de Pathogénie Microbienne Moléculaire, INSERM U1202, Institut Pasteur, Paris, France; 4 Research Unit on Obesity UR18ES01, Faculty of Medicine, University Tunis El Manar, Tunis, Tunisia; 5 Department of Nutritional Diseases A. National Institute of Nutrition and Food Technology, Tunis, Tunisia; 6 University of Carthage, National Institute of Applied Science and Technology, Tunis, Tunisia; 7 Chaire de Microbiologie et Maladies Infectieuses, Collège de France, Paris, France

**Keywords:** metataxonomic, microbiome, nutrition, Tunisian, Type 1 Diabetes, Type 2 Diabetes

## Abstract

Gut microbiota plays a key role in the regulation of metabolism and immunity. We investigated the profile of gut microbiota and the impact of dietary intake on gut bacterial distribution in diabetic and healthy Tunisian subjects, aiming to identify a dysbiotic condition, hence opening the way to restore eubiosis and facilitate return to health. In the present research, we enrolled 10 type 1 diabetic (T1D), 10 type 2 diabetic (T2D) patients and 13 healthy (H) subjects. Illumina Miseq technology was used to sequence V3-V4 hypervariable regions of bacterial 16SrRNA gene. Data were analyzed referring to QIIME 2 pipeline. RStudio software was used to explore the role of nutrition in gut bacterial distribution. At the phylum level, we identified an imbalanced gut microbiota composition in diabetic patients marked by a decrease in the proportion of *Firmicutes* and an increase in the abundance of *Bacteroidetes* compared with H subjects. We observed higher amounts of *Fusobacteria* and a decline in the levels of TM7 phyla in T1D patients compared with H subjects. However, we revealed a decrease in the proportions of *Verrucomicrobia* in T2D patients compared with H subjects. At the genus level, T2D subjects were more affected by gut microbiota alteration, showing a reduction in the relative abundance of *Faecalibacterium, Akkermansia, Clostridium, Blautia* and *Oscillibacter*, whereas T1D group shows a decrease in the proportion of *Blautia*. The gut bacteria distribution was mainly affected by fats and carbohydrates consumption. Gut microbiota composition was altered in Tunisian diabetic patients and affected by dietary habits.

## Introduction

Diabetes is a multifactorial chronic disease resulting from a complex interaction between genetic susceptibility and environmental factors [1] implicating altered gut microbial composition [2]. Whereas Type 1 diabetes results from autoimmune destruction of pancreatic β cells leading to insufficient insulin secretion, Type 2 diabetes is linked to insulin resistance associated to obesity [3]. The worldwide prevalence of diabetes in adults aged 20–79 years old was assessed to 10.5% [4] and 10.8% in Tunisia in 2021 [5].

Microbes coevolved with humans [6]. The gut microbiota is involved in food digestion, the production of vitamins [7], short chain fatty acids (SCFAs) [8], and the control of glucose [9] and bile acid. Alterations of the profile of gut microbiota affect metabolic and immune homeostasis [10] promoting the onset of diabetes and its complications. It was reported that T2D patients display a shift in their gut microbiota composition characterized by a decrease in microbial diversity compared with H subjects. Some butyrate producing bacteria are also reduced in subjects with T2D especially *Faecalibacterium prausnitzii* [11]. A decrease in the proportions of *Akkermansia* was also reported in this group [11]. This bacteria promotes the integrity of the mucin layer and impedes inflammatory mechanisms [11].

T1D patients revealed a dysbiotic gut microbiota, marked by a decrease of lactate producing bacteria as well as SCFAs producing bacteria [12].

Nutrition maintains healthy state of the host and develops the gut microbiota [13]. Fibers consumption enhances the growth and the diversity of bacteria that catabolize nutrients to produce SCFAs [14]. These molecules improve glucagon-like peptide-1 (GLP 1) elaboration leading to a better regulation of glycated hemoglobin (HbA1c) rates [14]. The composition of gut microbiota is affected by the quality of dietary fats ingested and high consumption of saturated fats, sugars and animal protein [15] altering therefore host metabolism [16]. In the present study, we investigated gut microbiota composition of Tunisian T1D and T2D patients and we assessed the impact of dietary habits on bacterial distribution, aiming to improve prevention and treatment of the diabetes by restoration of dysbiotic microbiota.

## Methods

### Clinical characteristics of the subjects

We recruited 20 diabetic patients (10 T1D and 10 T2D) and 13 H subjects aged from 19 to 66 years old from the National Institute of Nutrition and the Institute Pasteur in Tunis. All subjects signed written consent form to participate to the present study. Noting that in the present paper, we deciphered the gut bacterial profile of the same T1D and T2D patients enrolled in the research that we published in 2019 [17]. We used in the present study second generation sequencing technology to characterize the gut microbial landscape of this cohort targeting the hypervariable regions V3-V4 of the 16SrRNA gene rather than quantitative PCR (qPCR) approach as we did before. Diabetes was diagnosed referring to IDF criteria namely Fasting plasma glucose ≥ 7 mmol/L, HbA1c ≥ 6.5% [1].

Characteristics of subjects are indicated in Supplementary Table S1. As we mentioned in the table, the average age of T2D patients is 56.3 years. This ascertainment is in agreement with IDF report, certifying that Type 2 diabetes is commonly identified in elderly subjects [1].

H participants are not diabetic. Subjects under antibiotic and /or probiotic therapies within one month before stool sampling were excluded from the study. Stool samples were collected in sterile boxes and were frozen at −80°C.

### Microbial DNA extraction

We extracted from fecal samples 180-220 mg of microbial DNA using QiAamp Fast DNA Stool mini kit (51604, Qiagen). DNA concentrations were calculated using NanoDrop ND-2000 Spectrophotometer (ND-2000, Thermo Scientific). DNA was conserved at −20°C.

### Library preparation of 16S ribosomal DNA (16SrDNA) and V3-V4 sequencing

We amplified DNA (5ng/µl) extracted from feces using primers targeting 16SrDNA V3-V4. Amplification of the DNA was done using the forward 5′ TCGTCGGCAGCGTCAGATGTGTATAAGAGACAGCCTACGGGNGGCWGCAG 3′ and reverse 5′ GTCTCGTGGGCTCGGAGATGTGTATAAGAGACAGGACTACHVGGGTATCTAATCC 3′ primers including Illumina Overhang adapter sequences. PCR was run following those conditions 95°C for 3 min, 25 cycles of: 95°C for 30 s, 55°C for 30 s, 72°C for 30 s, with a final extension at 72°C for 5 min on a thermal cycler (GeneAmp PCR system 9700, Applied Biosystems), purification of amplicons was done using AMPure XP beads (A63881, Beckman Coulter). A second PCR was performed in order to attach dual indices and Illumina adapters sequences to the target 16SrDNA V3-V4 using the Nextera XT index kit. PCR was run following this program 95°C for 3 min 8 cycles of: 95°C for 30 s, 55°C for 30 s, 72°C for 30 s and final extension 72°C for 5 min. Amplicons were pooled in equimolar concentration and diluted in 10 mM Tris, pH 8.5, to obtain a final concentration of 2 nM. The pool was quantified and validated using Fragment Analyzer device (FSv2-CE2, Proteigene). Denaturation of pooled libraries was done with 0.2 N NaOH diluted with hybridization buffer, containing 10% of PhiX. Sequencing was performed on Illumina Miseq instrument (Miseq™2013) using MiSeq v3 reagent kits. A total of 11,076,396 sequencing reads were identified and 9,204,083 read pairs were generated. Analyses were performed using QIIME2 pipeline. Taxonomic assignment was achieved referring to the Human intestinal Data Base (HiTDB) version 1.0 [18]. We evaluated the alpha diversity referring to QIIME2 pipeline, statistical analysis were performed using Kruskal–Wallis test. Beta diversity was estimated using QIIME2 workflow and statistical analysis were achieved using permanova method. We evaluated the relative abundance of bacteria between groups Kruskal–Wallis test, we performed also pairwise analysis referring to Mann–Whitney test.

### Investigation of dietary consumption and its relation with gut bacteria distribution

We conducted weekly dietary surveys in order to evaluate food consumption of participants. Nutritional Data were analyzed by means of Nutrisoft-Tours Programme d'enquêtes alimentaires – Version 2.01 1988. Mann–Whitney test was used in order to assess nutrients consumption between diabetic patients and H subjects. Multivariate analysis was performed by means of RStudio. Spearman correlation test was used in order to assess differences between the consumption of nutrients and the distribution of gut bacteria in all subjects.

## Results

### Composition and abundance of gut bacteria in the study cohort

**Figure 1:**
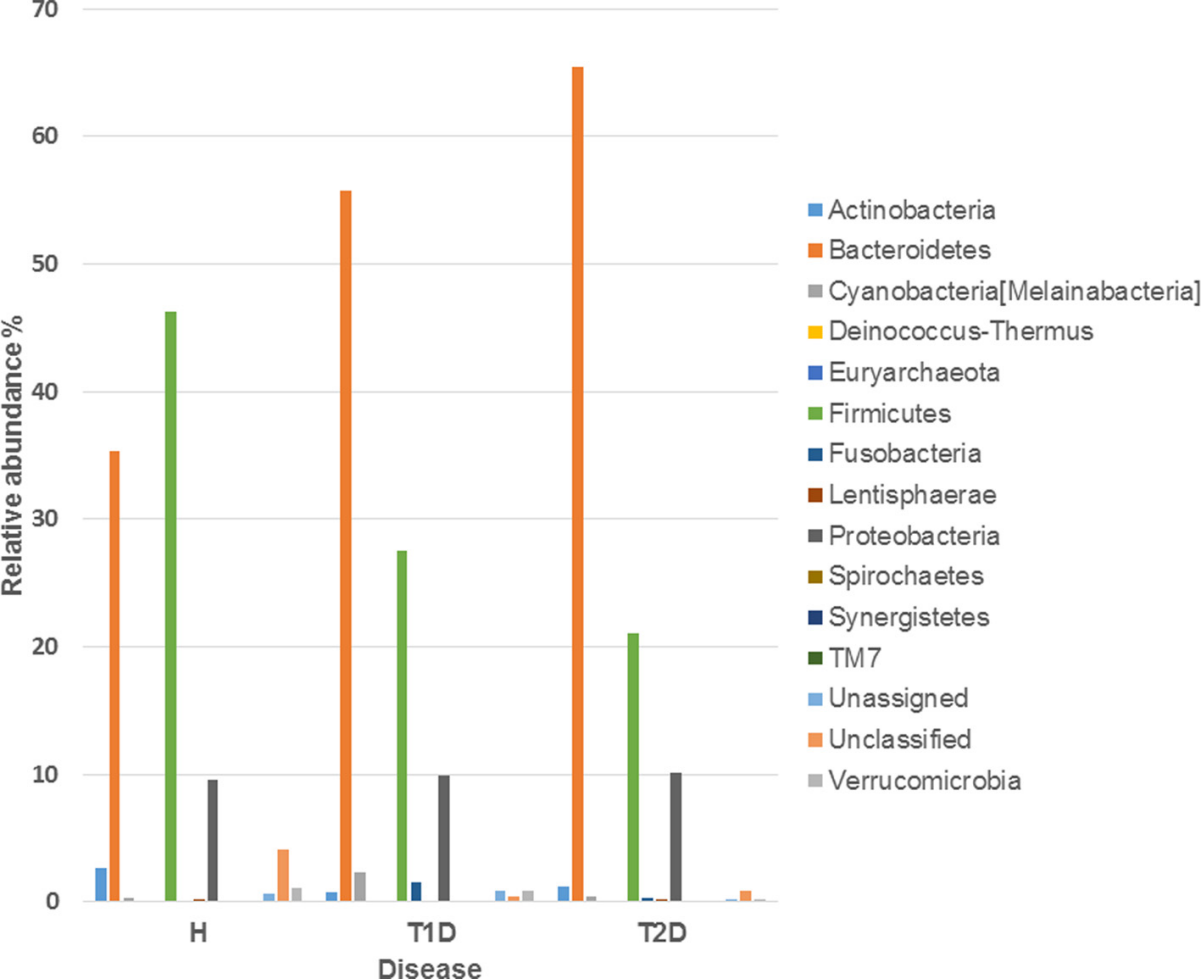
Relative abundance of gut bacteria in the investigated cohort H: healthy subjects, T1D: Type 1 diabetic patients, T2D: Type 2 diabetic patients.

**Figure 2:**
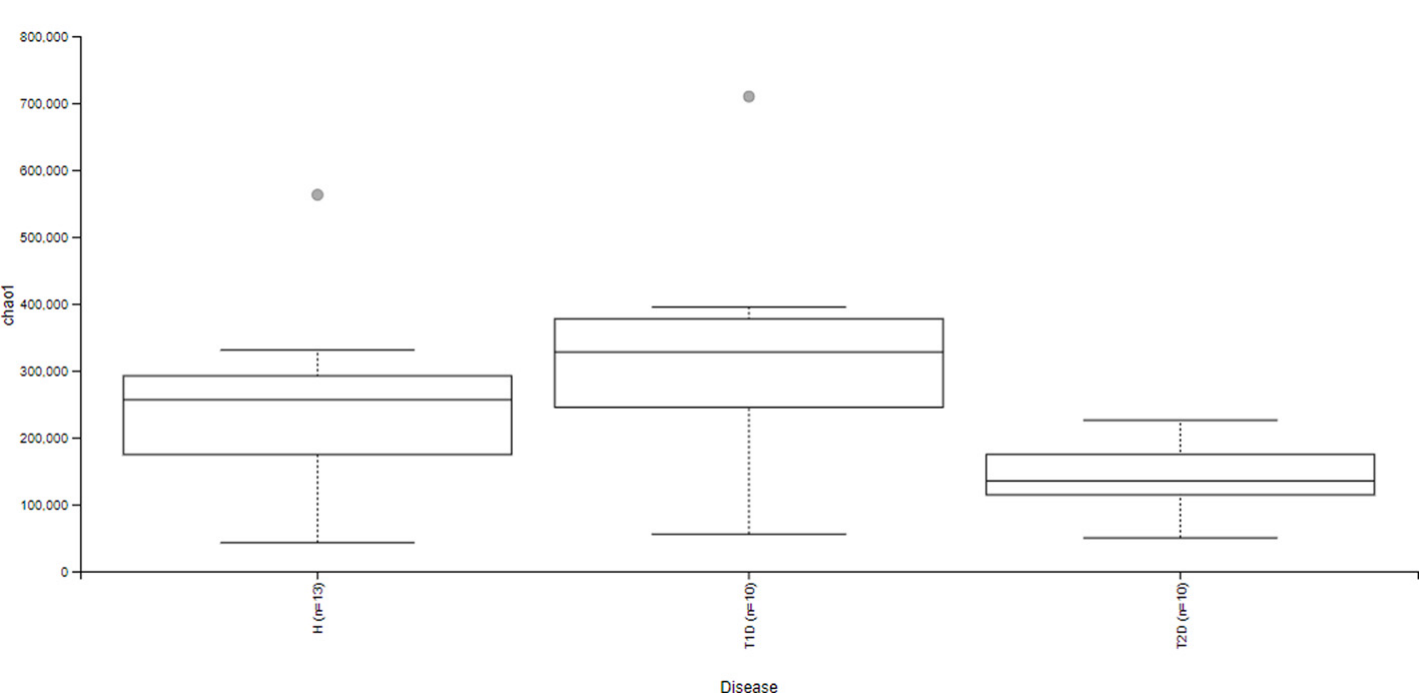
α-Diversity representation of gut bacteria in Tunisian subjects H: healthy subjects, T1D: Type 1 diabetic patients, T2D: Type 2 diabetic patients, *n*: number.

We previously analyzed by qPCR the bacterial species presented in the gut of diabetic patients and H Tunisian subjects [17], then, in order to get a more global vision, we sequenced V3-V4 hypervariables regions of the 16S rRNA gene using Illumina technology. At the phylum level, we detected a decrease in the relative abundance of Firmicutes between T1D patients (*P*=0.02) and T2D patients (*P*=0.0003) compared with H participants. We also observed an increase in the relative abundance of Bacteroidetes in diabetic groups compared with H subjects (*P*=0.001). We identified an increase in the proportions of Fusobacteria (*P*=0.01) and a reduction in the levels of TM7 (*P*=0.01) bacteria in T1D patients compared with H subjects. We detected a significant decrease in Verrucomicrobia in the gut microbiota composition of T2D patients compared with H subjects (*P*=0.01) (Figure 1). α-Diversity analysis reveals significant differences in the distribution of gut microbiota between T1D and T2D patients and H subjects (*P*=0.01) (Figure 2). Gut microbiota composition of T1D patients was more similar to H subjects compared with T2D patients. We showed that T2D patients had a low gut microbiota alpha diversity compared with T1D (*P*=0.01) patients and H subjects (*P*=0.009).

**Figure 3:**
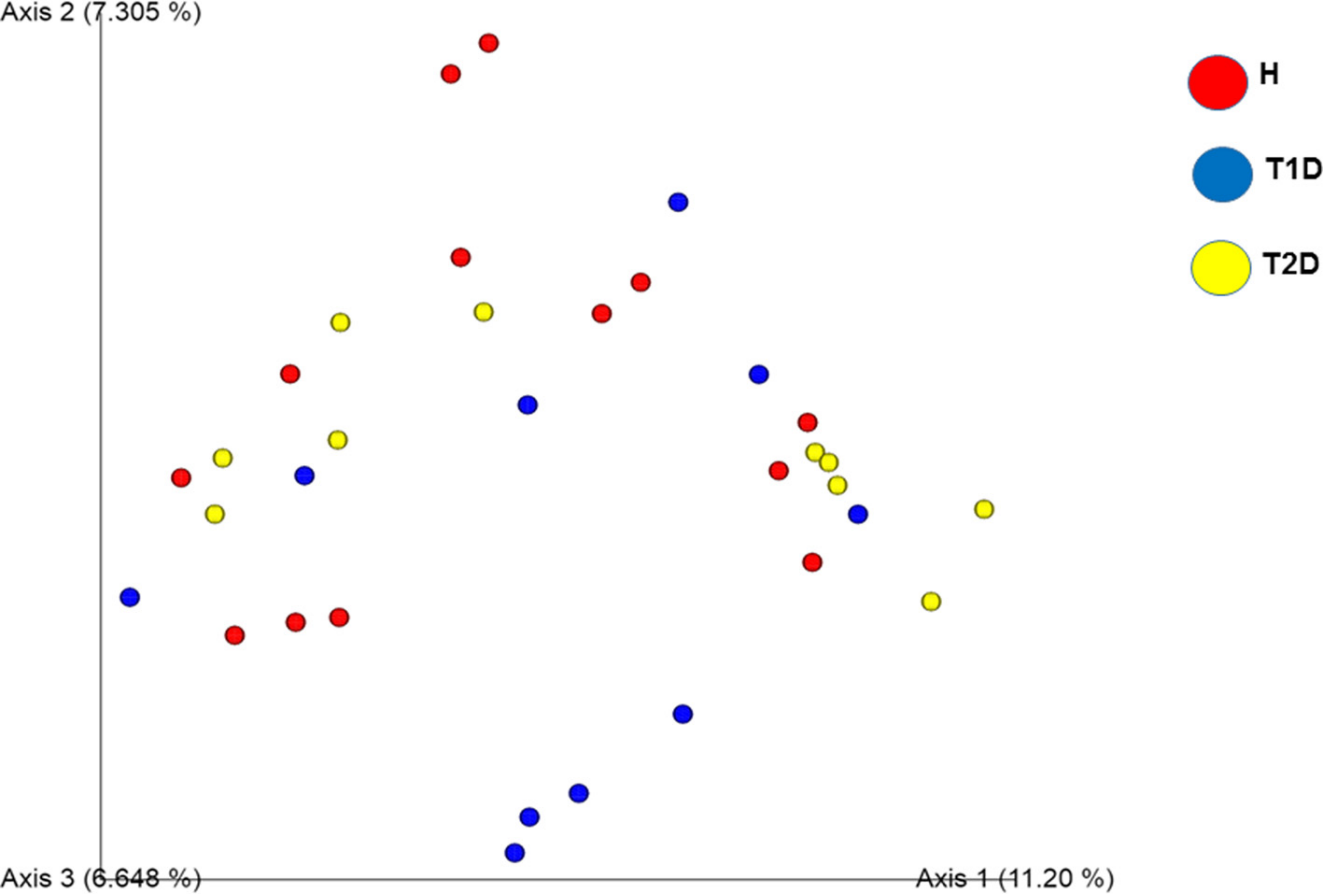
Principal coordinate analysis of gut bacteria in Tunisian participants H: healthy subjects, T1D: Type 1 diabetic patients, T2D: Type 2 diabetic patients.

The evaluation of the β-diversity showed that the gut microbiota composition of T2D patients was different from H subjects (*P*=0.02). Moreover, Principal coordinate analysis (PCoA) revealed that T2D and T1D patients formed different clusters, compared with H subjects (Figure 3).

**Figure 4:**
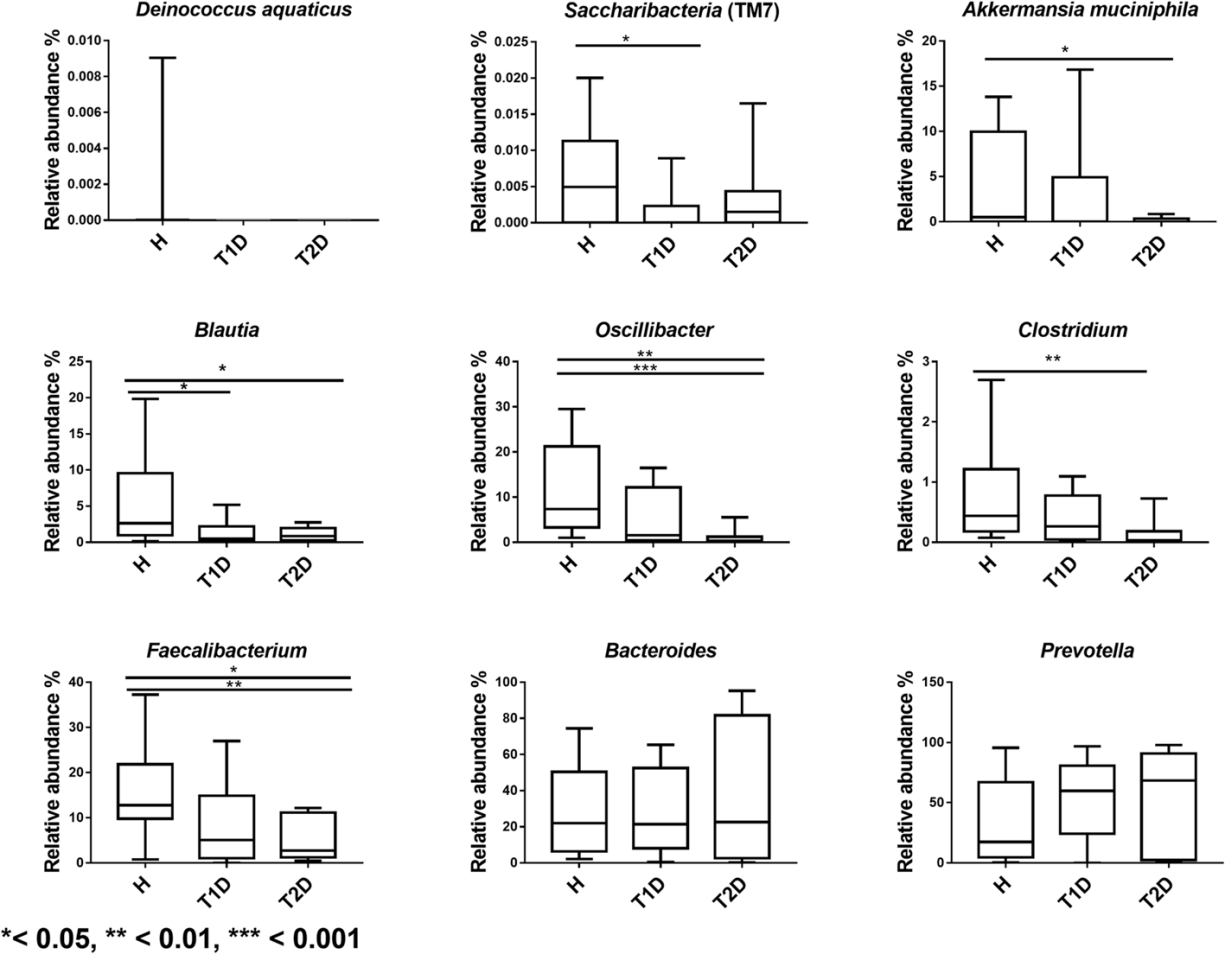
Boxplots representing the distribution of gut bacteria in Tunisian groups H: healthy subjects, T1D: Type 1 diabetic patients, T2D: Type 2 diabetic patients.

At the genus level, we detected a reduction in the proportions of *Oscillibacter* (*P*=0.0001), *Faecalibacterium* (*P*=0.005), *Akkermansia* (*P*=0.006), *Clostridium* (*P*=0.002), *Blautia* (*P* =0.02) in T2D patients compared with H subjects (Figure 4). A significant decline in the relative abundance of the phylum Saccharibacteria (TM7) (*P*=0.04), and a decrease in *Blautia* proportion (*P*=0.01) were also identified in the gut microbiome of T1D patients compared with H subjects (Figure 4).

### Correlation between nutrition and gut bacterial composition

The analysis of nutritional surveys showed that T1D patients consumed less saturated fatty acids (SFAs) (*P*=0.01) and more fibers (*P*=0.01) than H subjects. T2D patients consumed less SFAs (*P*=0.001), more fibers (*P*=0.03) and more vitamin C (*P*=0.03) than the H group (Supplementary Table S2).

**Figure 5:**
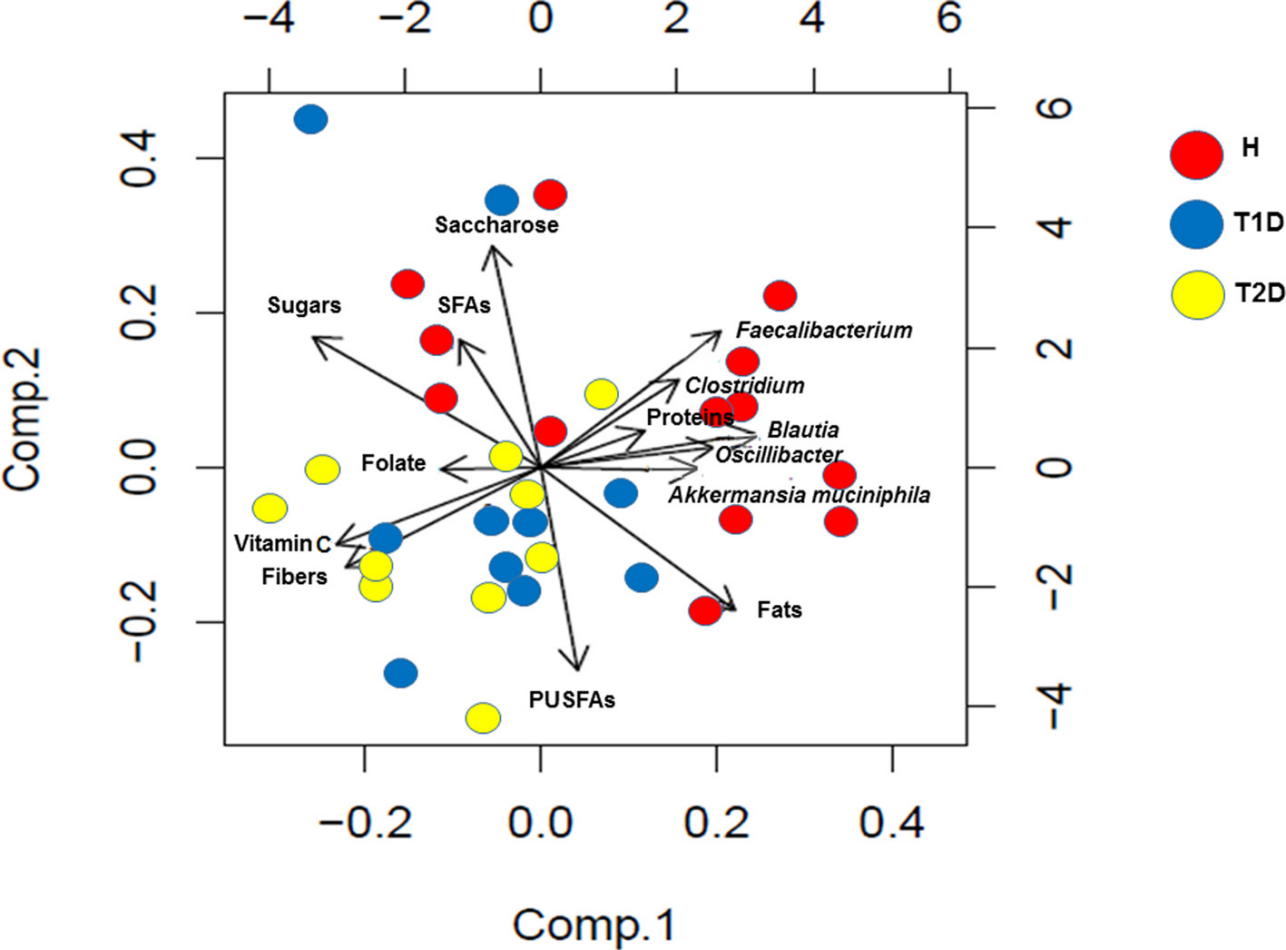
Dietary habits impact gut microbial distribution Comp.1: Component 1, Comp.2: Component 2, H: healthy subjects, T1D: Type 1 diabetic patients, T2D: Type 2 diabetic patients, SFAs: Saturated Fatty Acids, PUSFAs: Poly Unsaturated Fatty Acids.

Analysis of dietary surveys revealed a positive correlation between *Faecalibacterium* (*P*=0.01, rho = 0.41), *Oscillibacter* (*P*=0.01, rho = 0.41), *Clostridium* (*P*=0.04, rho = 0.35), *Blautia* (*P*=0.01, rho = 0.42) and the consumption of SFAs. Moreover saccharose intake positively correlated with the distribution of *Faecalibacterium* (*P*=0.03, rho = 0.36), and *Oscillibacter* (*P*=0.04, rho = 0.35). The consumption of fibers negatively correlated with the distribution of *Faecalibacterium* (*P*=0.02, rho = −0.39) and *Blautia* (*P*=0.01, rho = −0.41) (Figure 5). It was of notice that *Blautia* is the only bacteria which showed negative correlations with the consumption of sugars (*P*=0.002, rho = −0.51), vitamin C (*P*=0.01, rho = −0.41) and a positive association with fats intake (*P*=0.008, rho = 0.45). No significant correlations were identified between the distributions of *Saccharibacteria, Akkermansia* and the aforementioned nutrients (Figure 5).

## Discussion

This is a pilot study of gut microbiota composition of diabetic Tunisian patients compared with H subjects based on metataxonomic approach.

The composition of the gut microbiota is affected by different factors namely age, genetic, antibiotics, diet and lifestyle [19]. The age is not sufficient to explain the changes of gut microbiota profile and dysbiosis in T2D patients. In this context, it was demonstrated that the gut microbiota of centenarians individuals is characterized by an unforeseen proliferation of bacteria associated with healthy status and promoting a better metabolic and immune regulation, e.g., *Akkermansia* [19].

Altered gut microbiota profile of diabetic Tunisian subjects was associated with a decrease in the proportions of Firmicutes, Verrucomicrobiae, TM7 and an increase in the abundance of the Bacteroidetes and Fusobacteria phyla compared with H participants. Our results are in discordance with a study conducted on T2D Indian patients, revealing a reduction in the distribution of Bacteroidetes [20]. These discrepancies may be explained by the fact that gut microbiota composition differs between populations due to environmental factors, geographical location, cultural practices and diet [21]. We detected higher proportions of *Fusobacteria* and imbalanced distribution of TM7 in diabetic Tunisian patients. The increase in *Fusobacteria* [22] and an altered TM7 were associated to Inflammatory Bowel Disease (IBD) [23]. We detected a decrease of *Faecalibacterium, Blautia, Clostridium* and *Akkermansia* in T2D subjects. *Faecalibacterium* regulates energy intake [24] and intestinal gluconeogenesis [9]. Nonetheless, *Blautia* has been described as positively associated with T2D [25]. *Akkermansia muciniphila* holds α-glucosidase inhibitory activity preventing the catabolism of complex carbohydrates and decreasing postprandial hyperglycemia [25]. The gut microbiota ferments non digestible carbohydrates and produces saturated SCFAs including from 1 to 6 carbon atoms, represented respectively by Formate, acetate, propionate, butyrate, valerate and caproate [26]. Noting that *Faecalibacterium* produces butyrate [27]. This metabolite is recognized by G-protein coupled receptors (GPCR41+GPCR43) in intestine and improve the secretion of gut hormones GLP1, PYY, GLP2 [25]. This bacteria consolidates gut barrier via extracellular vesicles and enhances gut integrity, hampering consequently translocation of gut microbial antigens into systematic circulation and endotoxemia [25]. It was reported that *Akkermansia muciniphila* and *Blautia* produce acetate [26]. This metabolite is implicated in the regulation of carbohydrate and fat metabolism indeed SCFAs are able to modulate the immune and physiological functions of the host [26].

We revealed that diabetic Tunisian patients are consuming less healthy SFAs compared with H group. It was reported that SFAs provided by vegetables and fish consumption are considered as a main source of omega 3 Poly Unsaturated Fatty Acids (PUFAs), which promote microbial diversity [28]. However, SFAs provided by milk trigger inflammatory pathways [28]. Indeed, the intake of SFAs from dairy products contribute to complications, that could lead to metabolic dysfunction in diabetic Tunisian patients. We proved that carbohydrates intake (sugars, saccharose and fibers) modulate gut microbiota profile, this finding was also observed in recent study [29]. We observed that Tunisian T2D patients are consuming more vitamin C than H individuals. Ascorbic acid has antioxidant effects and has beneficial effects on gut bacteria [30]. T2D Tunisian patients have been under nutritional education and are followed by nutritionists, helping them to improve their dietary status that explain high vitamin C intake by T2D patients. In conclusion, our study highlights the altered diversity of gut microbiota in Tunisian diabetic patients compared with controls as well as the impact of dietary habits on gut microbial distribution. We recommend the identification of gut bacterial landscape during current diagnosis of diabetes in addition to the biochemical tests to guide targeted treatment of this disease and to promote precision medicine application. The restoration of dysbiotic gut bacteria with probiotics (based on the identification of gut bacterial biomarkers of healthy status) and prebiotics (balanced diet: vegetables and fruits) will stimulate the growth of beneficial bacteria and would ensure immune as well as metabolic adjustment. In perspective, we propose to decipher gut microbiota composition in a larger cohort.

## Supplementary Material

Supplementary Tables S1-S2

## Data Availability

Raw sequence data are available on Sequence Read Archive (SRA) under the BioProject PRJNA871997.

## Abbreviations

Β: Beta
BMI: Body Mass Index
°C: Degree Celsius
DNA: Deoxyribonucleic acid
GLP: glucagon-like peptide
GPCR: G-protein coupled receptors
H: Healthy
HbA1c: glycated hemoglobin A1c
HiTDB: Human intestinal Data Base
IBD: inflammatory bowel disease
IDF: International Diabetes Federation
mg: Milligram
ng: Nanogram
PUSFA: poly unsaturated fatty acid
PYY: Peptide YY
QIIME: Quantitative Insights Into Microbial Ecology
qPCR: quantitative polymerase chain reaction
rDNA: Ribosomal Deoxyribonucleic acid
rRNA: Ribosomal ribonucleic acid
SCFA: short chain fatty acid
SFA: saturated fatty acid
SRA: Sequence Read Archive
T1D: Type 1 diabetic
T2D: Type 2 diabetic
µl: Microliter
