# Supplementary material for: Gut microbiota profile and the influence of nutritional status on bacterial distribution in diabetic and healthy Tunisian subjects
Source: Biosci Rep. 2023 Sep 12;43(9):BSR20220803. doi: 10.1042/BSR20220803 (PMC10500226; doi:10.1042/BSR20220803)
Supplement: Supplementary Tables S1-S2 [file BSR-2022-0803_supp.pdf]

Supplementary Table S1. Anthropometric, biochemical and clinical characteristics of subjects.

Anthropometric and clinical characteristics	H (n = 13)	T1D (n = 10)	T2D (n = 10)
Gender	Eight females<br>Five males	Six females<br>Four males	Six females<br>Four males
Age (years) (mean ± SD)	31.07±7.06	27.9 ±9.02	56.3 ±9.85
BMIs (kg/m^2) (mean ± SD)	23.27±3.5	22.64 ± 3.72	28.77 ± 2.72
HbA1c (%) (mean ± SD)	5.23±0.67	8.7% ± 1.83	8.4% ±1.83
Fasting plasma glucose (mean mmol/l ± SD)	5.28±0.74	14.71 ± 5.98	11.10 ± 4

H: healthy subjects, T1D: type 1 diabetic patients, T2D: type 2 diabetic patients, SD: standard deviation, BMIs: Body mass indexes, HbA1C: glycated haemoglobin.

Supplementary Table S2. Estimations of the proportions of daily nutrients consumption in the cohort

Energetic and Macronutrients	H (n= 13) Means ±SD	T1D patients (n = 10) Means ±SD	T2D patients (n = 10) Means ±SD	T1D patients versus H p-values	T2D patients versus H p-values
Daily energetic intake (Kcal/day)	2368,23 ± 440.40	2255,88 ± 472.65	2300 ± 496.10	0.39	0.52
Digestible carbohydrates (%)	49.60 ± 6.23	52.01 ± 4.68	52.85 ± 4.06	0.26	0.21
Fats (%)	38.2 ± 5.98	36.97 ± 4.61	35.2 ± 4.53	0.79	0.21
Saturated fatty acids (%)	13.16 ± 2.68	10.30 ± 2.51	8.85 ± 2.13	0.017	0.001
Mono unsaturated fatty acids (%)	18.14 ± 3.82	17.26 ± 4.16	18.10 ± 3.64	0.84	0.83
Poly unsaturated fatty acids (%)	6.88 ± 2.80	9.40 ± 4.09	8.23 ± 4.46	0.051	0.41
Proteins (%)	12.2 ± 1.75	11.01 ± 1.66	11.95 ± 1.91	0.21	0.74

Micronutrients	H (n= 13) Means ±SD	T1D patients (n = 10) Means ±SD	T2D patients (n = 10) Means ±SD	T1D patients versus H p-values	T2D patients versus H p-values
Zinc (mg/day)	10.33 ± 2.86	8.30 ± 1.96	9.94 ± 1.59	0.06	0.56
Calcium (mg/day)	673.07 ± 318.16	580.33 ± 259.70	662.2 ± 181.67	0.51	1
Iron (mg/day)	8.44 ± 1.78	8.98 ± 1.92	7.76 ± 1.51	0.51	0.37
Sodium (mg/day)	1780 ± 563.07	2037.11 ± 820.28	1910 ± 976.43	0.51	0.97
Potassium (mg/day)	2288.07 ± 729.36	2664.11 ± 574.30	2870.8 ± 1245.66	0.12	0.23
Phosphore (mg/day)	1156.46 ± 269.90	1068.77 ± 208.98	1093.8 ± 257.03	0.51	0.44
Fibers (g/day)	12.75 ± 6.51	20.17 ± 5.96	20.81 ± 9.77	0.014	0.036

Vitamin B1 (mg/day)	0.70 ± 0.18	0.88 ± 0.43	0.58 ± 0.18	0.64	0.15
Vitamin C (mg/day)	44.76 ± 36.98	70.88 ± 43.61	95.4 ± 58.22	0.11	0.03
Folates (µg/day)	160.51 ± 61.35	217.72 ± 67.04	154.94 ± 50.74	0.06	0.97
